# *Apolipoprotein E* E3/E4 genotype is associated with an increased risk of coronary atherosclerosis in patients with hypertension

**DOI:** 10.1186/s12872-024-04169-3

**Published:** 2024-09-12

**Authors:** Guoliang Wei, Bin Li, Hao Wang, Li Chen, Wenhao Chen, Kehui Chen, Weihong Wang, Shen Wang, Hui Zeng, Yuanliang Liu, Yue Zeng, Hui Rao

**Affiliations:** 1 Center for Cardiovascular Diseases, Meizhou People’s Hospital, Meizhou Academy of Medical Sciences, Meizhou, China; 2 Data Center, Meizhou People’s Hospital, Meizhou Academy of Medical Sciences, Meizhou, China; 3 Department of Laboratory Medicine, Meizhou People’s Hospital, Meizhou Academy of Medical Sciences, Meizhou, China

**Keywords:** Coronary atherosclerosis, Hypertension, *APOE*, Susceptibility

## Abstract

**Objective:**

*Apolipoprotein E* (*APOE*) gene polymorphisms were associated with coronary atherosclerosis and hypertension. However, the relationship between *APOE* polymorphisms and coronary atherosclerosis susceptibility in hypertensive patients is unclear. The aim of this study was to assess the relationship.

**Methods:**

A total of 1713 patients with hypertension who were admitted to Meizhou People’s Hospital from November 2019 to August 2023 were retrospectively analyzed, including 848 patients with coronary atherosclerosis and 865 patients without coronary atherosclerosis. The rs429358 and rs7412 polymorphisms of *APOE* were genotyped, and relationship between *APOE* polymorphisms and the risk of coronary atherosclerosis in hypertensive patients were analyzed.

**Results:**

There were 10 (0.6%), 193 (11.3%), 30 (1.8%), 1234 (72.0%), 233 (13.6%), and 13 (0.8%) individuals with *APOE* ɛ2/ɛ2, ɛ2/ɛ3, ɛ2/ɛ4, ɛ3/ɛ3, ɛ3/ɛ4, and ɛ4/ɛ4 genotype, respectively. The frequency of *APOE* ɛ3/ɛ4 was higher (16.4% vs. 10.9%, *p* = 0.001) in the patients with coronary atherosclerosis than controls. Logistic analysis showed that body mass index (BMI) ≥ 24.0 kg/m^2^ (24.0 kg/m^2^ vs. 18.5–23.9 kg/m^2^, odds ratio (OR): 1.361, 95% confidence interval (CI): 1.112–1.666, *p* = 0.003), advanced age (≥ 65/<65, OR:1.303, 95% CI: 1.060–1.602, *p* = 0.012), history of smoking (OR: 1.830, 95% CI: 1.379–2.428, *p* < 0.001), diabetes mellitus (OR: 1.380, 95% CI: 1.119–1.702, *p* = 0.003), hyperlipidemia (OR: 1.773, 95% CI: 1.392–2.258, *p* < 0.001), and *APOE* ɛ3/ɛ4 genotype (ɛ3/ɛ4 vs. ɛ3/ɛ3, OR: 1.514, 95% CI: 1.133–2.024, *p* = 0.005) were associated with coronary atherosclerosis in hypertensive patients.

**Conclusions:**

Overweight (BMI ≥ 24.0 kg/m^2^), advanced age, history of smoking, diabetes mellitus, and *APOE* ɛ3/ɛ4 genotype were independent risk factors for coronary atherosclerosis in hypertensive patients.

## Introduction

Hypertension is a cardiovascular disease characterized by continuous elevation of systolic and/or diastolic blood pressure (SBP/DBP) in systemic arteries, and it is also an influential factor for a variety of serious cardiovascular and cerebrovascular diseases [1]. Hypertension is a worldwide chronic non-communicable disease, a major disease that endangers human health, and the leading cause of global disease burden [2]. In China, the standardized prevalence of hypertension in adults aged 18–69 is about 24.7% [3]. With the progress of population aging, the prevention and treatment of hypertension in China is facing great challenges [4–6]. Cardiovascular disease (CVD) is the leading cause of death worldwide, accounting for more than 7 million deaths each year [7]. Coronary atherosclerosis is a heart disease caused by myocardial ischemia or necrosis due to stenosis, blockage and spasm of coronary artery atherosclerosis [8, 9]. Coronary atherosclerosis is a multi-cause disease, a range of risk factors for coronary atherosclerosis, including age, gender, high blood pressure, smoking, and diabetes mellitus have been identified in Framingham Heart Study [10]. The disease has become a common disease in Europe countries and the United States [11]. And the incidence of the disease has also shown a significant increase in China in the past 10 years [12]. Major risk factors for coronary atherosclerosis include diabetes mellitus, hypertension, smoking, and obesity [13, 14].

It is not uncommon for hypertension and coronary atherosclerosis to occur in the same person at the same time, and studies have shown that people with hypertension have a higher tendency to develop coronary atherosclerosis than those with normal blood pressure [15, 16]. In a cohort study based on the electronic health records of 1.25 million adults, the risk of death from coronary atherosclerosis increased by 26% for every 20mmHg increase in unadjusted SBP [17]. The prevalence rate of hypertension combined with coronary atherosclerosis is increasing year by year [18, 19]. These two diseases share some common etiological factors, such as obesity, inflammation, oxidative stress, and microvascular and macrovascular damage [13, 20]. The treatment of patients with hypertension combined with coronary atherosclerosis is a challenge. Therefore, predicting the risk of coronary atherosclerosis in hypertensive patients may be more beneficial to the treatment and control of these diseases [21].

Lipid metabolism disorders are closely related to CVDs including hypertension and coronary atherosclerosis, and apolipoproteins play an important role in lipid metabolism. As an apolipoprotein, apolipoprotein E (ApoE) plays an important role in lipid metabolism, lipid transport and distribution, and immune regulation [22–25]. ApoE binds to cholesterol receptors to mediate cholesterol metabolism in the liver, and also acts as a cofactor to mediate the degradation of lipoproteins by lipid metabolizing enzymes [26, 27]. The function of APOE is affected by the rs429358 and rs7412 polymorphisms of the *APOE* gene [27]. Based on the variants of these two polymorphisms, APOE can form three alleles (ε2, ε3 and ε4) and 6 genotypes (ɛ2/ɛ2, ɛ2/ɛ3, ɛ2/ɛ4, ɛ3/ɛ3, ɛ3/ɛ4, and ɛ4/ɛ4) [28–31]. The differences in the receptor binding capacity and the rate of mediating lipoprotein metabolism of APOE encoded by different *APOE* genotypes are the mechanisms of the different lipid metabolism regulatory capacity between different *APOE* variants [32]. The relationship between *APOE* gene polymorphisms and coronary atherosclerosis susceptibility in hypertensive population is unclear. The purpose of this study was to investigate this relationship.

## Materials and methods

### Study participants and data collection

This study retrospectively analyzed 1713 patients with hypertension who were admitted to Meizhou People’s Hospital from November 2019 to August 2023. Inclusive criteria of hypertensive patients were the following: (1) a mean SBP > 140 mmHg and/or a mean DBP > 90 mmHg [33], (2) Age ≥ 18 years old. Among these hypertensive patients, 848 patients with coronary atherosclerosis served as the study group and 865 patients without coronary atherosclerosis as the control group. The diagnostic criteria for coronary atherosclerosis: Coronary angiography (CAG) showed that at least one of the main epicardial vessels (including left main branch, anterior descending branch, circumflex branch, and right coronary artery) had a diameter stenosis, and clinically diagnosed myocardial infarction [34, 35]. Information such as age, sex, body mass index (BMI), history of smoking, history of alcohol consumption, history of diabetes mellitus, and history of hypertension were collected from the patient’s medical record information system. According to the height and weight standards of the Chinese population, BMI was divided into three grades: <18.5 kg/m^2^, 18.5–23.9 kg/m^2^, and ≥ 24.0 kg/m^2^ [36, 37]. A diagnosis of hyperlipidemia can be made if one of the following conditions is met: (1) total cholesterol (TC) ≥ 6.22 mmo1/L), (2) triglyceride (TG) ≥ 2.26 mmo1/L, or (3) low-density lipoprotein-cholesterol (LDL-C) ≥ 4.14 mmo1/L, according to the Guidelines for the Prevention and Control of Dyslipidemia in Chinese Adults [38, 39].

### Determination of serum lipids and *APOE* genotyping

Fasting blood was collected and serum was isolated. Total cholesterol (TC), triglyceride (TG), high-density lipoprotein cholesterol (HDL-C), and low-density lipoprotein cholesterol (LDL-C), Apolipoprotein A1 (Apo-A1), and Apolipoprotein B (ApoB) levels in serum samples were assessed using automatic biochemical analysis system (Olympus AU5400 system, Tokyo, Japan) and corresponding kits.

Genomic DNA was extracted from venous blood collected from EDTA anticoagulant collection vessels using a blood DNA isolation kit (Qiagen GmbH, Germany). The quality and concentration of the DNA were assessed using a Nano-Drop 2000™ spectrophotometer (ThermoFisher Scientific, USA). *APOE* genotype was amplified by PCR - microarray method (Sinochips Bioscience Co., Ltd., China).

### Statistical analysis

Continuous variables were expressed as means ± standard deviations and were compared using either Student’s t-test or the Mann-Whitney U test. The Hardy-Weinberg equilibrium analysis of the subjects, and the comparison of genotype composition ratio and allele frequency between the two groups were analyzed using χ^2^ test. Univariate analysis and multivariate regression logistic analysis were applied to examine the relationship between *APOE* rs429358 and rs7412 genotypes and alleles and coronary atherosclerosis in patients with hypertension. *p* < 0.05 was considered to represent statistical significance. All statistical analysis were performed using SPSS statistical software version 26.0 (IBM Inc., USA).

## Results

### Characteristics of subjects

Among the subjects included in this study, 1071 (62.5%) men and 642 (37.5%) women had hypertension. There were 624 (36.4%) hypertensive patients with < 65 years old and 1089 (63.6%) patients with ≥ 65 years old. About half of the subjects (50.4%) had BMI within the normal range (18.5–23.9 kg/m^2^), and 767 (44.8%) were overweight (≥ 24 kg/m^2^). There were 334 (44.8%) patients had history of smoking, 79 (4.6%) patients had history of alcohol consumption, and 561 (32.7%) patients with diabetes mellitus, respectively. There was no significant difference in gender distribution, age distribution, and history of alcohol consumption between patients with coronary atherosclerosis and controls (all *p* > 0.05). In the controls, there were 45 (5.2%), 468 (54.1%), and 352 (40.7%) patients with BMI < 18.5 kg/m^2^, 18.5–23.9 kg/m^2^, and ≥ 24.0 kg/m^2^, respectively. There were 38 (4.5%) patients with coronary atherosclerosis had BMI < 18.5 kg/m^2^ and 415 (48.9%) had BMI ≥ 24.0 kg/m^2^. The proportion of BMI ≥ 24.0 kg/m^2^ in patients with coronary atherosclerosis was higher than that in controls (48.9% vs. 40.7%, *p* = 0.003). The proportion of history of smoking (23.7% vs. 15.4%, *p <* 0.001), diabetes mellitus (37.0% vs. 28.6%, *p <* 0.001), and hyperlipidemia (27.2% vs. 16.5%, *p <* 0.001) in patients with coronary atherosclerosis was higher than that in controls, respectively. The patients with coronary atherosclerosis had higher TC (4.57 ± 1.20 vs. 4.46 ± 1.03 mmol/L, *p* = 0.043) and TG (1.86 ± 1.55 vs. 1.51 ± 0.87 mmol/L, *p* < 0.001), and lower HDL-C (1.18 ± 0.34 vs. 1.23 ± 0.38 mmol/L, *p* = 0.002) levels than controls (Table 1).

**Table 1:** Clinical characteristics of patients with hypertension in this study

Variables	Total (*n* = 1713)	Controls (*n* = 865)	Patients with coronary atherosclerosis (*n* = 848)	*p* values
Gender				
Male, n(%)	1071(62.5%)	535(61.8%)	536(63.2%)	0.562
Female, n(%)	642(37.5%)	330(38.2%)	312(36.8%)	
Age, years				
< 65, n(%)	624(36.4%)	336(38.8%)	288(34.0%)	0.036
≥ 65, n(%)	1089(63.6%)	529(61.2%)	560(66.0%)	
BMI (kg/m^2^)				
< 18.5, n(%)	83(4.8%)	45(5.2%)	38(4.5%)	0.003
18.5–23.9, n(%)	863(50.4%)	468(54.1%)	395(46.6%)	
≥ 24.0, n(%)	767(44.8%)	352(40.7%)	415(48.9%)	
History of smoking				
No, n(%)	1379(80.5%)	732(84.6%)	647(76.3%)	< 0.001
Yes, n(%)	334(19.5%)	133(15.4%)	201(23.7%)	
History of alcohol consumption				
No, n(%)	1634(95.4%)	830(96.0%)	804(94.8%)	0.260
Yes, n(%)	79(4.6%)	35(4.0%)	44(5.2%)	
Diabetes mellitus				
No, n(%)	1152(67.3%)	618(71.4%)	534(63.0%)	< 0.001
Yes, n(%)	561(32.7%)	247(28.6%)	314(37.0%)	
Hyperlipidemia				
No, n(%)	1339(78.2%)	722(83.5%)	617(72.8%)	< 0.001
Yes, n(%)	374(21.8%)	143(16.5%)	231(27.2%)	
Serum lipid-lipoprotein levels				
TC, mmol/L	4.51 ± 1.12	4.46 ± 1.03	4.57 ± 1.20	0.043
TG, mmol/L	1.69 ± 1.26	1.51 ± 0.87	1.86 ± 1.55	< 0.001
HDL-C, mmol/L	1.21 ± 0.36	1.23 ± 0.38	1.18 ± 0.34	0.002
LDL-C, mmol/L	2.44 ± 0.81	2.43 ± 0.77	2.46 ± 0.85	0.387
Apo-A1, g/L	1.09 ± 0.27	1.10 ± 0.28	1.09 ± 0.26	0.395
Apo-B, g/L	0.79 ± 0.23	0.78 ± 0.22	0.80 ± 0.25	0.020

BMI, body mass index; TC, total cholesterol; TG, triglycerides; HDL-C, high-density lipoprotein-cholesterol; LDL-C, low-density lipoprotein-cholesterol; Apo-A1, apolipoprotein A1; Apo-B, apolipoprotein B

### Distribution frequencies of *APOE* genotypes and alleles in patients with coronary atherosclerosis and controls

The results of χ^2^ test showed that the *APOE* rs429358 and rs7412 in the patients with coronary atherosclerosis (*χ*^2^ = 0.797, *p* = 0.372; and *χ*^2^ = 0.0003, *p* = 0.986), and controls (*χ*^2^ = 2.094, *p* = 0.148; and *χ*^2^ = 0.448, *p* = 0.503) conformed to the Hardy-Weinberg equilibrium, respectively. There were 10 (0.6%), 193 (11.3%), 30 (1.8%), 1234 (72.0%), 233 (13.6%), and 13 (0.8%) individuals with *APOE* ɛ2/ɛ2, ɛ2/ɛ3, ɛ2/ɛ4, ɛ3/ɛ3, ɛ3/ɛ4, and ɛ4/ɛ4 genotype, respectively. The frequency of the *APOE* ɛ3/ɛ3 genotype was lower (69.7% vs. 74.3%, *p* = 0.036), and ɛ3/ɛ4 genotype was higher (16.4% vs. 10.9%, *p* = 0.001) in the patients with coronary atherosclerosis than those in controls. The frequency of the ε3 allele was lower (83.3% vs. 85.7%, *p* = 0.048), while ε4 higher (9.9% vs. 7.0%, *p* = 0.003) in the patients with coronary atherosclerosis than those in controls (Table 2).

**Table 2:** Distribution frequencies of *APOE* genotypes and alleles in patients with coronary atherosclerosis and control

Variable	Genotype/allele	Total (*n* = 1713)	Controls (*n* = 865)	Patients with coronary atherosclerosis (*n* = 848)	χ^2^	*p* values
*APOE* rs429358						
	T/T	1437(83.9%)	751(86.8%)	686(80.9%)	11.120	0.001
	T/C	263(15.4%)	107(12.4%)	156(18.4%)	11.966	0.001
	C/C	13(0.8%)	7(0.8%)	6(0.7%)	0.059	0.808
HWE (χ^2^, *p*)		χ^2^ = 0.064, *p* = 0.800	χ^2^ = 2.094, *p* = 0.148	χ^2^ = 0.797, *p* = 0.372		
*APOE* rs7412						
	C/C	1480(86.4%)	744(86.0%)	736(86.8%)	0.222	0.637
	C/T	223(13.0%)	115(13.3%)	108(12.7%)	0.118	0.731
	T/T	10(0.6%)	6(0.7%)	4(0.5%)	0.363	0.547
HWE (χ^2^, *p*)		χ^2^ = 0.257, *p* = 0.612	χ^2^ = 0.448, *p* = 0.503	χ^2^ = 0.0003, *p* = 0.986		
*APOE* genotype						
	ɛ2/ɛ2	10(0.6%)	6(0.7%)	4(0.5%)	0.363	0.753
	ɛ2/ɛ3	193(11.3%)	102(11.8%)	91(10.7%)	0.482	0.493
	ɛ2/ɛ4	30(1.8%)	13(1.5%)	17(2.0%)	0.627	0.465
	ɛ3/ɛ3	1234(72.0%)	643(74.3%)	591(69.7%)	4.580	0.036
	ɛ3/ɛ4	233(13.6%)	94(10.9%)	139(16.4%)	11.121	0.001
	ɛ4/ɛ4	13(0.8%)	7(0.8%)	6(0.7%)	0.059	1.000
*APOE* allele						
	ε2	243(7.1%)	127(7.3%)	116(6.8%)	0.327	0.595
	ε3	2894(84.5%)	1482(85.7%)	1412(83.3%)	4.761	0.048
	ε4	289(8.4%)	121(7.0%)	168(9.9%)	9.399	0.003

HWE, Hardy Weinberg Equilibrium

### Clinical characteristics of subjects stratified by *APOE* ɛ2, ɛ3, ɛ4 alleles

Because the ɛ2 and ɛ4 alleles have opposite functions in lipid metabolism, individuals carrying ɛ2/ɛ4 genotypes (*n* = 30) were not included in the analysis of the relationship between *APOE* alleles and clinical characteristics of patients in this study. Clinical characteristics and serum lipid-lipoprotein levels were compared among all subjects carried different *APOE* alleles. There was no significant difference in gender distribution, age distribution, proportion of history of smoking, history of alcohol consumption, and diabetes mellitus between patients with coronary atherosclerosis and controls (all *p* > 0.05). The level of LDL-C (2.27 ± 0.88 vs. 2.46 ± 0.84 and 2.63 ± 0.83 mmol/L, *p* = 0.006) was lower in the patients with *APOE* ɛ2 allele than those with ɛ3 and ɛ4 allele, respectively (Table 3).

**Table 3:** Clinical characteristics of patients with coronary atherosclerosis stratified by *APOE* ɛ2, ɛ3, ɛ4 alleles

Variables	ε2 (ɛ2/ɛ2 + ɛ2/ɛ3) (*n* = 95)	ε3 (ɛ3/ɛ3) (*n* = 591)	ε4 (ɛ3/ɛ4 + ɛ4/ɛ4) (*n* = 145)	*p* values
Gender				
Male, n(%)	55(57.9%)	373(63.1%)	97(66.9%)	0.371 (χ^2^=2.003)
Female, n(%)	40(42.1%)	218(36.9%)	48(33.1%)	
Age, years				
< 65, n(%)	28(29.5%)	207(35.0%)	50(34.5%)	0.575 (χ^2^=1.122)
≥ 65, n(%)	67(70.5%)	384(65.0%)	95(65.5%)	
BMI (kg/m^2^)				
< 18.5, n(%)	5(5.3%)	27(4.6%)	5(3.4%)	0.966 (χ^2^=0.585)
18.5–23.9, n(%)	42(44.2%)	272(46.0%)	67(46.2%)	
≥ 24.0, n(%)	48(50.5%)	292(49.4%)	73(50.3%)	
History of smoking				
No, n(%)	74(77.9%)	451(76.3%)	108(74.5%)	0.824 (χ^2^=0.390)
Yes, n(%)	21(22.1%)	140(23.7%)	37(25.5%)	
History of alcohol consumption				
No, n(%)	93(97.9%)	555(93.9%)	139(95.9%)	0.219 (χ^2^=3.062)
Yes, n(%)	2(2.1%)	36(6.1%)	6(4.1%)	
Diabetes mellitus				
No, n(%)	60(63.2%)	383(64.8%)	82(56.6%)	0.182 (χ^2^=3.410)
Yes, n(%)	35(36.8%)	208(35.2%)	63(43.4%)	
Hyperlipidemia				
No, n(%)	57(60.0%)	446(75.5%)	102(70.3%)	0.005 (χ^2^=10.423)
Yes, n(%)	38(40.0%)	145(24.5%)	43(29.7%)	
Serum lipid-lipoprotein levels				
TC, mmol/L	4.63 ± 1.22	4.52 ± 1.20	4.72 ± 1.19	0.197
TG, mmol/L	2.20 ± 1.71*	1.80 ± 1.57^#^	1.87 ± 1.26	0.062
HDL-C, mmol/L	1.22 ± 0.35	1.18 ± 0.35	1.17 ± 0.32	0.502
LDL-C, mmol/L	2.27 ± 0.88*^,$^	2.46 ± 0.84^#,$^	2.63 ± 0.83^#,^*	0.006
Apo-A1, g/L	1.14 ± 0.24*	1.08 ± 0.27^#^	1.08 ± 0.26	0.076
Apo-B, g/L	0.75 ± 0.28^$^	0.81 ± 0.25	0.85 ± 0.23^#^	0.018

BMI, body mass index; TC, total cholesterol; TG, triglycerides; HDL-C, high-density lipoprotein-cholesterol; LDL-C, low-density lipoprotein-cholesterol; Apo-A1, apolipoprotein A1; Apo-B, apolipoprotein B

^#^compared with ε2, *p* < 0.05

*compared with ε3, *p* < 0.05

^$^compared with ε4, *p* < 0.05

### Logistic regression analysis of risk factors of coronary atherosclerosis in patients with hypertension

In univariate analysis, BMI ≥ 24.0 kg/m^2^ (BMI ≥ 24.0 kg/m^2^ vs. BMI 18.5–23.9 kg/m^2^, odds ratio (OR): 1.397, 95% confidence interval (CI): 1.149–1.698, *p* = 0.001), history of smoking (Yes vs. No, OR: 1.710, 95% CI: 1.341–2.181, *p* < 0.001), diabetes mellitus (Yes vs. No, OR: 1.471, 95% CI: 1.201–1.802, *p* < 0.001), hyperlipidemia (Yes vs. No, OR: 1.890, 95% CI: 1.495–2.390, *p* < 0.001), *APOE* ɛ3/ɛ4 genotype (ɛ3/ɛ4 vs. ɛ3/ɛ3, OR: 1.609, 95% CI: 1.210–2.138, *p* = 0.001), and *APOE* ɛ4 allele (ɛ4 vs. ɛ3, OR: 1.562, 95% CI: 1.183–2.061, *p* = 0.002) were significantly associated with coronary atherosclerosis in patients with hypertension. In multivariate regression logistic analysis, advanced age (≥ 65/<65, OR: 1.303, 95% CI: 1.060–1.602, *p* = 0.012), BMI ≥ 24.0 kg/m^2^ (≥ 24.0 kg/m^2^ vs. 18.5–23.9 kg/m^2^, OR: 1.361, 95% CI: 1.112–1.666, *p* = 0.003), history of smoking (Yes vs. No, OR: 1.830, 95% CI: 1.379–2.428, *p* < 0.001), diabetes mellitus (Yes vs. No, OR: 1.380, 95% CI: 1.119–1.702, *p* = 0.003), hyperlipidemia (Yes vs. No, OR: 1.773, 95% CI: 1.392–2.258, *p* < 0.001), and *APOE* ɛ3/ɛ4 genotype (ɛ3/ɛ4 vs. ɛ3/ɛ3, OR: 1.514, 95% CI: 1.133–2.024, *p* = 0.005) were independent risk factors for coronary atherosclerosis in patients with hypertension (Table 4).

**Table 4:** Logistic regression analysis of risk factors of coronary atherosclerosis in patients with hypertension

Variables	Univariate OR (95% CI)	*p* values	Multivariate OR (95% CI)	*p* values
Gender (Female/Male)	0.944 (0.776–1.148)	0.562	1.080 (0.869–1.344)	0.487
Age (≥ 65/<65, years)	1.235 (1.014–1.504)	0.036	1.303 (1.060–1.602)	0.012
BMI (kg/m^2^)				
18.5–23.9	1.000 (reference)	-	1.000 (reference)	-
< 18.5	1.001 (0.637–1.572)	0.998	1.069 (0.674–1.696)	0.776
≥ 24.0	1.397 (1.149–1.698)	0.001	1.361 (1.112–1.666)	0.003
History of smoking (Yes/No)	1.710 (1.341–2.181)	< 0.001	1.830 (1.379–2.428)	< 0.001
History of alcoholism (Yes/No)	1.298 (0.824–2.044)	0.261	0.905 (0.549–1.492)	0.696
Diabetes mellitus (Yes/No)	1.471 (1.201–1.802)	< 0.001	1.380 (1.119–1.702)	0.003
Hyperlipidemia (Yes/No)	1.890 (1.495–2.390)	< 0.001	1.773 (1.392–2.258)	< 0.001
*APOE* genotypes				
ɛ3/ɛ3	1.000 (reference)	-	1.000 (reference)	-
ɛ2/ɛ2	0.725 (0.204–2.583)	0.620	0.732 (0.200-2.676)	0.637
ɛ2/ɛ3	0.971 (0.716–1.315)	0.848	0.893 (0.653–1.221)	0.478
ɛ2/ɛ4	1.423 (0.685–2.954)	0.344	1.383 (0.653–2.931)	0.397
ɛ3/ɛ4	1.609 (1.210–2.138)	0.001	1.514 (1.133–2.024)	0.005
ɛ4/ɛ4	0.933 (0.312–2.791)	0.901	0.879 (0.284–2.720)	0.822

BMI, body mass index

## Discussion

Hypertension is a chronic disease based on vascular disease, and the long-term increase of blood pressure is easy to cause damage to some organs and cause corresponding diseases [40, 41]. Hypertension is closely related to coronary atherosclerosis and is a risk factor for coronary atherosclerosis [42, 43]. The increase of pulse pressure can increase the tension of artery vessels, increase the tension of artery wall, fatigue and even fracture of elastic fibers, damage vascular endothelial cells, and accelerate or promote the development of atherosclerosis [44]. Prospective cohort studies have shown a significantly increased risk of CVD in people with higher baseline blood pressure [45]. Foreign cohort studies have shown that changes in individual blood pressure level or progression of blood pressure classification are also associated with the long-term or lifelong risk of CVDs [46, 47]. Studies have shown that hypertension is prone to co-exist with metabolic diseases such as obesity, abnormal blood sugar and dyslipidemia, and these metabolic diseases often become an important cause of the occurrence and death of CVDs [48]. The course of disease is a multi-factorial process of change. It is of great significance to understand the risk of cardiovascular and cerebrovascular events in hypertensive patients based on genetics and personal habits. In patients with hypertension, the risk factors for CAD remain unclear. Therefore, it is of great significance to identify the risk of CAD in hypertensive patients. In this study, overweight (BMI ≥ 24.0 kg/m^2^), history of smoking, diabetes mellitus, *APOE* ɛ3/ɛ4 genotype, and *APOE* ɛ4 allele were independent risk factors for coronary atherosclerosis in patients with hypertension.

*APOE* polymorphism is associated with an increased risk of coronary artery disease in Finnish adults [49]. Some studies found that *APOE* ε4 allele may be associated with an increased risk of CAD in Egyptians [50], ethnic Kashmiri population [51], and Chinese populations [52, 53], respectively. Balcerzyk et al. found that the synergistic effect of the ε4 allele with some traditional risk factors (such as smoking, high cholesterol levels) is associated with an increased risk of CAD [54]. Other studies have found that *APOE* ɛ4 allele was associated with an increased risk of diabetes mellitus complicated with CAD [31, 55]. In addition, some studies have found that *APOE* polymorphisms are also associated with the severity of coronary atherosclerosis [56, 57]. However, some studies have found that *APOE* polymorphisms were not associated with the susceptibility of coronary atherosclerosis [58, 59].

As an indicator of the degree of obesity, studies have found that when BMI is maintained in the normal range, the occurrence of coronary atherosclerosis can be reduced [60]. Previous studies have shown that high BMI is associated with an increased risk of CVDs [61–63]. Some studies found that the risk of major adverse cardiovascular events was not affected by BMI [64, 65]. Chen et al. found that BMI ≥ 24.0 kg/m^2^ was an independent risk factor for type 2 diabetes mellitus (T2DM) complicated with CAD [31]. In addition, BMI is associated with long-term prognosis and risk of death from CAD [66, 67]. BMI is different in population due to gender, age and race, and has certain limitations in risk prediction [68]. Therefore, comprehensive attention should be paid to the role of some risk factors in coronary atherosclerosis susceptibility in people with hypertension.

Hypertensive people with a history of smoking have a higher risk of major cardiovascular events. CVD accounts for about one third of smoking-related deaths worldwide [69]. Study has found that there is a dose-effect relationship between smoking and CVDs, and long-term small smoking will also increase the risk of CVDs [70]. The mechanism of the potential association between tobacco and CVD may be due to harmful chemicals in tobacco that promote the development of CVD by increasing heart rate, heart muscle contraction, inflammation, endothelial damage, thrombosis, and affecting lipid levels [71]. In this study, univariate regression analysis showed that smoking was a risk factor for coronary atherosclerosis in hypertensive individuals, but multivariate regression logistic analysis did not obtain this result. However, in any case, health education should be strengthened for hypertensive patients and smoking cessation intervention should be carried out to reduce the risk of coronary atherosclerosis.

This study is the first to report on *APOE* gene polymorphisms and coronary atherosclerosis susceptibility in hypertensive patients. There are some inadequacies in this study. First, this study retrospectively collected data from the medical record system of the included subjects, and did not include other possible influencing factors of coronary atherosclerosis for analysis. Second, the subjects included in this study are all patients seeking treatment in Meizhou People’s Hospital, so the selection of subjects may be biased. Third, the relationship between *APOE* gene polymorphisms and the risk of coronary atherosclerosis in patients with different grades of hypertension has been not analyzed in this study.

## Conclusion

In summary, overweight (BMI ≥ 24.0 kg/m^2^), advanced age, history of smoking, diabetes mellitus, and *APOE* ɛ3/ɛ4 genotype were independent risk factors for coronary atherosclerosis in patients with hypertension. It means that hypertensive patients who are obesity, have history of smoking and diabetes mellitus, and carried the *APOE* ɛ3/ɛ4 genotype need to be aware of the risk of developing coronary atherosclerosis.

## Data Availability

No datasets were generated or analysed during the current study.
